# Stretching the Spines of Gymnasts: A Review

**DOI:** 10.1007/s40279-015-0424-6

**Published:** 2015-11-18

**Authors:** William A. Sands, Jeni R. McNeal, Gabriella Penitente, Steven Ross Murray, Lawrence Nassar, Monèm Jemni, Satoshi Mizuguchi, Michael H. Stone

**Affiliations:** Centre for Sport and Exercise Science, Sheffield Hallam University, Sheffield, UK; 2300 South 2100 East, Salt Lake City, UT 84109 USA; Department of Physical Education, Health and Recreation, Eastern Washington University, Cheney, WA USA; Academy of Sport and Physical Activity, Sheffield Hallam University, Sheffield, UK; Department of Kinesiology, Colorado Mesa University, Grand Junction, CO USA; MSU Sports Medicine, Michigan State University, East Lansing, MI USA; Department of Sport Science, College of Arts and Sciences, Qatar University, Doha, Qatar; Department of Physical Education, Exercise and Sport, East Tennessee State University, Johnson City, TN USA

## Abstract

Gymnastics is noted for involving highly specialized strength, power, agility and flexibility. Flexibility is perhaps the single greatest discriminator of gymnastics from other sports. The extreme ranges of motion achieved by gymnasts require long periods of training, often occupying more than a decade. Gymnasts also start training at an early age (particularly female gymnasts), and the effect of gymnastics training on these young athletes is poorly understood. One of the concerns of many gymnastics professionals is the training of the spine in hyperextension—the ubiquitous ‘arch’ seen in many gymnastics positions and movements. Training in spine hyperextension usually begins in early childhood through performance of a skill known as a back-bend. Does practising a back-bend and other hyperextension exercises harm young gymnasts? Current information on spine stretching among gymnasts indicates that, within reason, spine stretching does not appear to be an unusual threat to gymnasts’ health. However, the paucity of information demands that further study be undertaken.

## Key Points

**Table:** 

Spinal flexibility, particularly flexibility of the lumbar spine, is an important ability in young female gymnasts and is trained intensively from very young ages.
Spine stretching in gymnastics training may be an important contributor to spinal abnormalities, injury and pain, demanding careful progression and vigilant monitoring of the development of young gymnasts.
Spine stretching and loading among gymnasts should be undertaken via careful, thorough and long-term progression.

## Introduction

Shawn Johnson, an American gold-medallist in the Beijing Olympics, remarked in the US media that she was not sure if she would allow her own daughter to participate in gymnastics, and she described the sport as brutal [1]. Questions have arisen and remain regarding appropriate training regimens for young gymnasts [2–5]. Extreme ranges of motion (ROMs) and the contortionist-like positions of the spine performed by gymnasts have produced reactionary discourse among physicians, fans, coaches, athletes, parents and scientists [6–11]. Can gymnastics training, specifically spine hyperextension stretching, be inappropriate for young gymnasts? In particular, do specific skills and positions—such as the back-bend, bridge, backward and forward bends or limbers, backward and forward walkovers, and backward and forward flic-flacs or handsprings—subject the gymnast to unusual and dangerous injury threats [12]? Is there reason to believe that spine stretching should be delayed, modified or abandoned? Is there a threshold age and/or ability that must be reached prior to safe performance of spine stretching? If so, how would one know this threshold? Can specialized training, particularly focused on spine stretching, be both a cause and countermeasure for the development of spinal injuries as the young gymnast grows and matures [12]?

Gymnastics is an ‘early’ sport, with the majority of training occurring prior to adulthood [2, 3, 13–15]. However, few studies have targeted preadolescent athletes [5, 16]. Longitudinal data are especially sparse on spinal ‘extreme stretching’. Most stretching studies have involved short-term, cross-sectional, pre- and post-test ‘snapshots’ of stretching interventions. These studies have usually sought to compare different stretching programmes and their influence on changes in ROM [6, 17–19]. As such, assessment of stretching effects in growing children is particularly difficult [4, 5]. The lack of long-term studies ensures that delayed effects of training will likely remain unknown [4, 20]. Long-term studies are also needed to identify threshold ages or sensitive ages for spine stretching. Causes and mechanisms are often blurred by extraneous variables from growth and maturation, and the intrusion of other day-to-day training factors, such as injury. Observing gymnasts and measuring aspects of stretching over long periods likely fits the definition of a ‘natural experiment’ as described by Susser [21]. Natural experiments, such as gymnastics stretching, may inform investigations in lieu of laboratory and other controlled studies.

Stretching is the method for which flexibility is the outcome. Stretching is the elongation of the muscle-and-tendon complex by application of a force or torque that places the muscle-and-tendon complex at its maximum length [22]. Flexibility is defined as the pain-free ROM of a joint or a related series of joints [22–24]. Mobility is an expansion of the concept of flexibility, adding fluidity and coordinated ease of motion [25]. Hypermobility, for our purposes, refers to a condition involving extreme flexibility, due in part to a genetic anomaly that influences the extensibility and elasticity of connective tissue [26]. Stretching, flexibility, mobility and hypermobility are not the same thing, and each requires an appropriate definition for understanding of the available literature [19, 22, 23, 26–30]. A theoretical framework for this topic will apply the definitions above within the following components: gymnastics training, growth and heritability, spinal alignment, joint hypermobility syndrome, contortionism, yoga, spinal injury, and risk management and countermeasures. This review provides the most current information on spine hyperextension flexibility for all stakeholders in gymnastics, with the objective of improving training methods and decisions via enhanced knowledge.

## Gymnastics Training

Gymnasts often begin participation in early childhood with specialization soon afterward [31, 32]. The nature of acrobatic skills requires spine mobility, and serious stretching often begins as young as 4 or 5 years [33–35]. There is a paucity of research specifically addressing flexibility in young children (i.e. 4–11 years), with the amount of literature increasing in direct proportion to age [30]. In a study by Bruggemann [36], female gymnasts between the ages of 12 and 13 years showed the highest incidence of spinal abnormalities. Prepubertal and peripubertal gymnasts have served as discrete samples representing ‘young’ gymnasts [29, 37–39]. Unfortunately, the era of training, gymnast’s age, training age, competitive levels, and volume and intensity factors have been inconsistently included in gymnastics investigations. Further complicating any understanding of the flexibility of young gymnasts is the fact that stretching interacts with many other aspects of gymnastics training [40].

Gymnastics is not a static sport; its rules, interpretations and fashions change rapidly and systematically. The changing milieu of gymnastics results in varying training demands for different ages and abilities [41–44]. Physical fitness, energetic demands, and strength and power requirements have been described by several investigators [45–48]. Historical gymnastics fitness profiles over several decades have shown that the demands on gymnasts have increased in parallel with the progressive rules changes as established by the gymnastics Code of Points [49]. The Code of Points, without being a gymnastics coaching manual, drives much of gymnastics training [45, 46, 50]. Each country uses the Code of Points for international competition and often modifies the international rules for lower-level domestic competitions and training. The Code of Points changes almost continuously via rule interpretations, with large changes occurring at least following each Olympiad. The emphasis on flexibility was more prominent in earlier Codes. The current demands of gymnastics require less emphasis on extreme ROMs in poses, postures and skills, while increasing the physical demands for strength and stability of the spine. The changing demands from the Code of Points nearly always trickle down to the lower competitive levels, including young children. Spinal loads from extreme ROMs have reduced the emphasis on simple static poses emphasizing spinal flexibility in recent years. Slow-moving spine hyperextension and flexion motions, such as forward and backward walkovers, are rarely observed, except in lower-level compulsory routines. Modern gymnastics tends to emphasize high-speed extension and flexion motions, which are parts of skills such as the spine hyperextension in Yurchenko vault preflights, Tkatchev flight phases on the uneven bars and landings involving partially completed somersaults and twists [51–53].

**Fig. 1:**
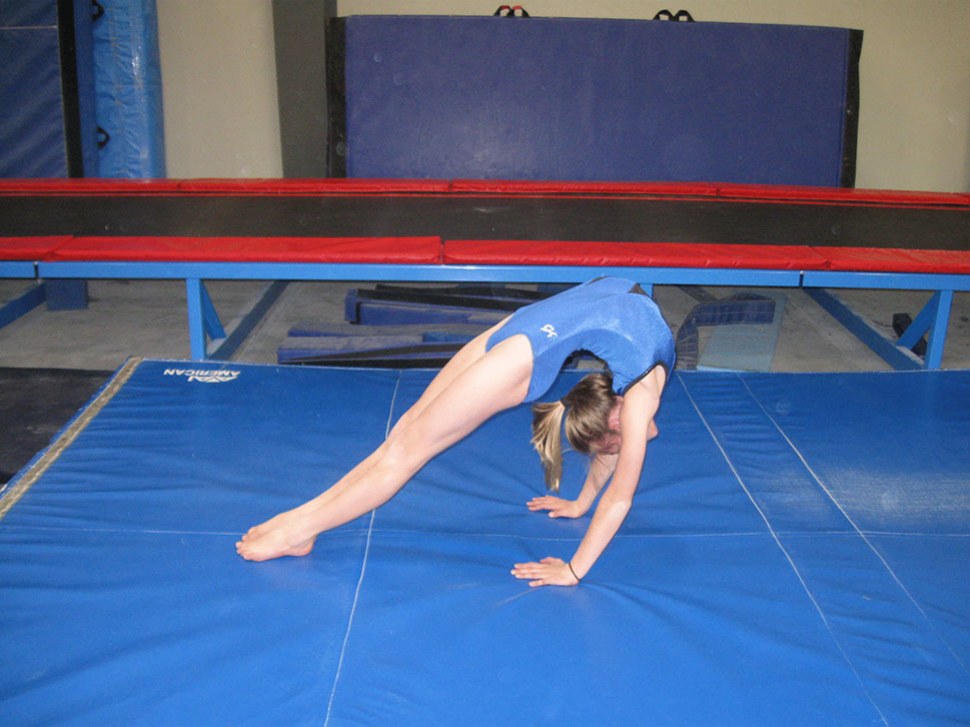
Back-bend position emphasizing hyperextension in the thoracic spine and shoulder hyperflexion

**Fig. 2:**
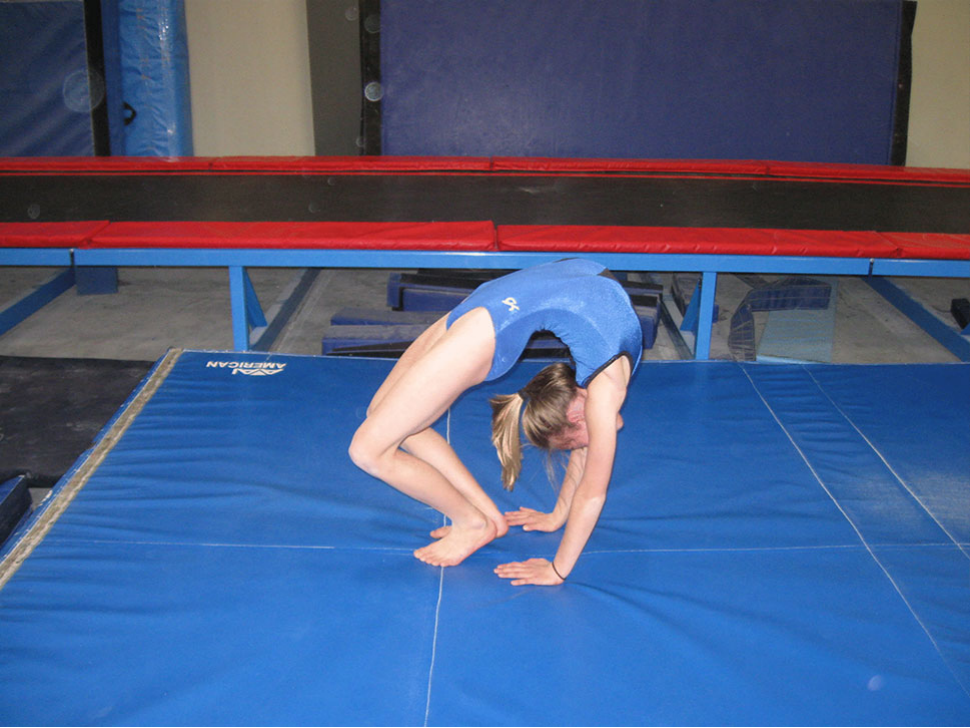
Back-bend position emphasizing hyperextension in the lumbar spine

In addition to variations in time, gymnastics often uses multiple terms to refer to the same skill, and terms tend to move in and out of common usage. For example, the terms ‘back-bend’ and ‘bridge’ are sometimes synonymous. A back-bend has also been described as lowering rearward from a stand by hyperextension of the spine and hips to contact the floor or apparatus with the hands [54]. A bridge usually refers to a static position of the spine and hip in hyperextension, with weight supported on the hands and feet. There are different styles of back-bends in performance, based on the placement of the majority of spine hyperextension. Figure 1 shows a back-bend position emphasizing hyperextension in the thoracic spine and shoulder hyperflexion. Figure 2 shows a back-bend position that emphasizes stretching of the lumbar spine by emphasizing the hands being close to the feet. These positions and classifications based on gymnastics skill names are naïve. In unloaded positions, such as those in Figs. 1 and 2, the gymnast may confine the majority of stretching to a portion of the spine. However, when the gymnast moves to and from these positions, the majority of the hyperextension may shift dynamically from one area of the spine to another. Moreover, the spine is a three-dimensional structure, which bends dynamically with natural curves in the sagittal (flexion/extension), frontal (lateral flexion) and horizontal (twisting left or right) planes. The combination of these curves result in a spine that spirals [55–63].

**Fig. 3:**
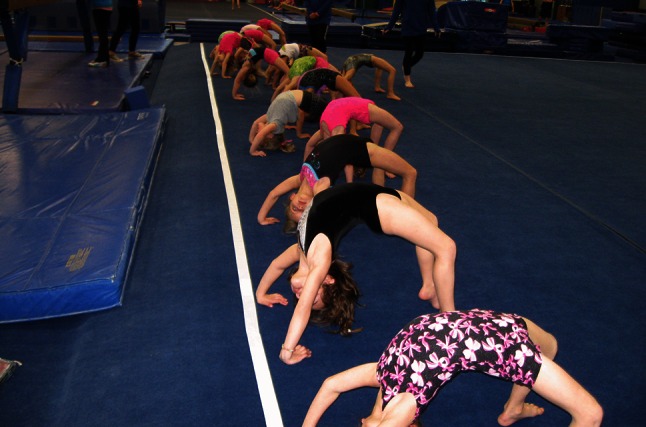
Young gymnasts performing warmup stretching of their spines. Note the poor positions and the lack of emphasis on placing the majority of the spinal extension in the shoulders and upper back

Modern approaches to spine stretching in gymnastics encourage the position in Fig. 1, while discouraging the position in Fig. 2 [12, 54, 64–66]. However, too often, young gymnasts are not supervised with sufficient attention to detail (Fig. 3) and are allowed to perform spine-stretching exercises that result in poor positions that remain uncorrected. Sadly, the early exposures to spine-stretching movements and positions are often habituated through repetition and must be corrected later, with a considerable investment in skill re-education, time and adjustment of positions. Too often, the early learning habits acquired from this type of training are never completely extinguished in later training and become manifest when the young gymnast attempts new skills or novel movements, or is placed under competitive stress.

**Fig. 4:**
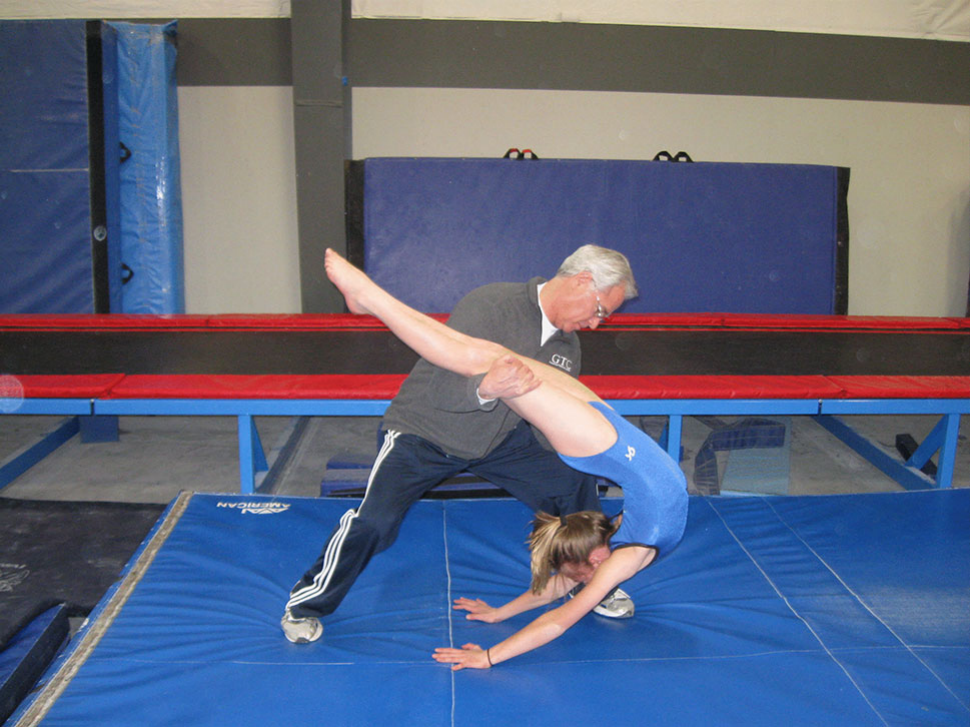
Exaggerated position emphasizing shoulder and upper torso stretch

**Fig. 5:**
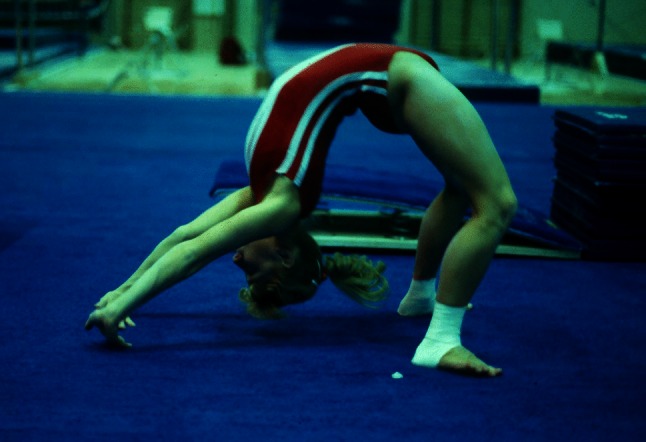
Back-bend position using the lumbar spine in a back-bend. Note that the shoulders show extension such that the head is exposed to striking the floor

Moving to and from the back-bend position requires a dynamic spine hyperextension. In all such movements, the hyperextension should begin with shoulder hyperflexion (the arms moving behind the head) and superior spine hyperextension. When the gymnast begins to lower backward from a stand to a back-bend, the superior spine hyperextension begins at the superior torso and proceeds incrementally from the most superior to the most inferior vertebrae. When lowering to a back-bend from a handstand, the gymnast again begins the movement in the shoulders and superior spine [54]. Figure 4 shows an exaggerated position emphasizing the nature of the motion during lowering from a handstand to the back-bend position. Figure 5 shows a staged back-bend position emphasizing stretching the lumbar spine, while neglecting the superior spine and shoulders. In spite of numerous educational resources, including the Talent Opportunity Program physical ability test procedures [67], books [64, 66, 68] and articles [54, 64], many coaches appear to disregard the importance of back-bend technique.

## Growth and Heritability

Flexibility is a morphological characteristic governed largely by heritable characteristics, and it is known to rely on multiple physical, genetic and motor control factors. Flexibility is considered joint specific and motion specific [23].

Bouchard et al. [69] indicated heritability values of 0.69 for low back flexibility in 11- to 15-year-old males and 0.84 for trunk flexibility in male and female twins aged 12–17 years. Other heritability values for flexibility were 0.84 for the trunk, 0.70 for the hip and 0.91 for the shoulder. Bouchard et al. [69] concluded that genetics may have a more powerful influence on flexibility than on strength. It has been shown that joint hypermobility can be passed from parents to children [70].

Muscle stiffness and reflex magnitudes, along with muscle spindle sensitivity, are known to increase with age in 7- to 11-year-olds, while remaining below adult capabilities. Muscle co-activation is greater in children and declines with age [71–73]. The period of peak height velocity has been postulated as a period when flexibility is reduced or plateaus [74, 75]. It has also been suggested that flexibility is reduced during this period because bone growth outpaces muscle lengthening [3, 14]. Long-term athlete development programmes have suggested critical periods for flexibility development, particularly between the ages of 6 and 11 years [76, 77].

In terms of non-gymnast schoolchildren, cross-sectional investigations began at least as early as the 1940s. Gurewitsch and O’Neil [78] conducted one of the earliest studies on children and showed declining flexibility from 6 to 12 years of age, followed by slight increases in ROM up to 18 years. Purcell and Micheli [106] studied 4500 children from kindergarten to 12th grade (i.e. senior high school) on two flexibility tests. The tests included a simple standing toe-touch and lowering the forehead to the knees while seated with straight legs. At the age of 5 years, 98 % of the boys and 86 % of the girls could perform the toe-touch, but, by the age of 6 years, flexibility had already declined. By the age of 12 years, only 30 % of both sexes could successfully perform the toe-touch test. A ‘pulse’ of improved flexibility occurred between approximately 13 and 17 years of age. The more extreme flexibility test—touching the forehead to the knees—resulted in 15 % of the girls and 5 % of the boys initially achieving the position, and these percentages remained stable through the age of 17 years. The age-related sensitive periods for flexibility development as described by Gurewitsch and O’Neil [78] (i.e. sit and reach) are supported by Drabik [33], Alter [23] and Bouchard et al. [69]. There is a paucity of cross-sectional and longitudinal studies of flexibility among young athletes. Sands [29] studied active and passive flexibility of US gymnasts from the age of 9 years through the senior national team (aged >15 years). Sands showed that shoulder hyperflexion and superior spinal ROM improved with age in US female gymnasts from 9 years of age to the senior national team [29]. Studies focusing specifically on spinal flexibility in young athletes are rare.

## Spinal Alignment

**Fig. 6:**
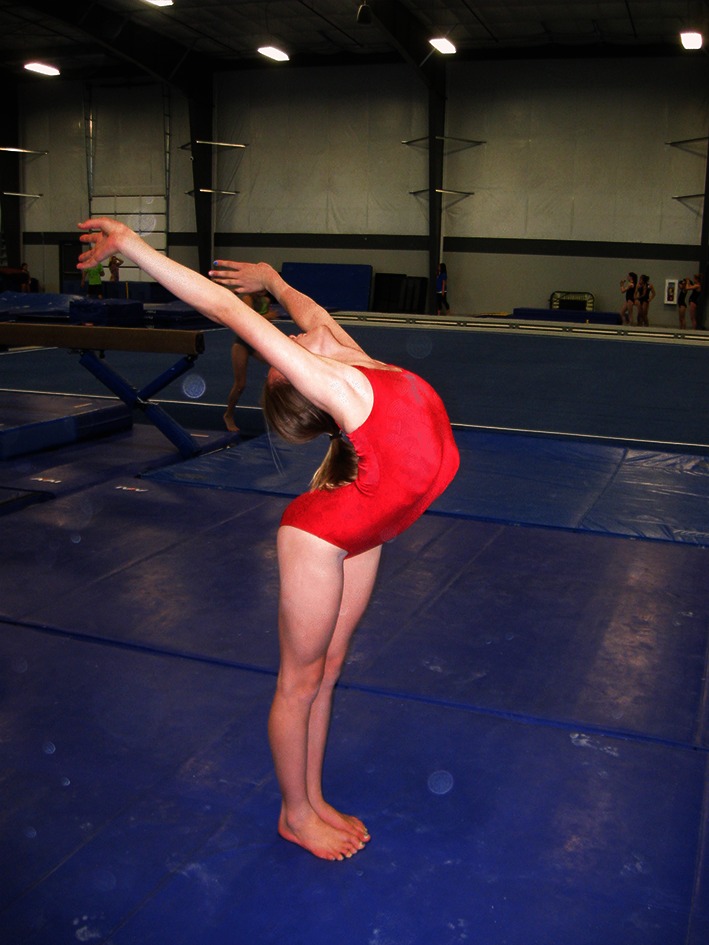
Signature position when ending a routine. The gymnast shows a hyperextended cervical, thoracic and lumbar spine. The hyperextension is particularly dramatic in the lumbar spine

Gymnasts, particularly female artistic gymnasts, often display a signature movement at the dismount ending a routine (Fig. 6). The gymnast stands with arms raised triumphantly overhead, with the cervical and lumbar spines greatly hyperextended. A study of sagittal plane spinal curvatures in 64 female gymnasts with a mean age of 12 years showed that one degree of total sagittal lumbar ROM was lost for every degree of increased lordosis. Twenty percent of the girls reported low back pain in conjunction with the greatest lordosis [79]. A radiographic and magnetic resonance imaging (MRI) study of 35 young gymnasts and ten control subjects showed that in spite of excessive ROMs and high axial loading, damage to the intervertebral discs was uncommon during growth [80]. A large study of 2270 children in many sports (407 girls and 1863 boys) between 8 and 18 years of age showed that thoracic kyphosis and lumbar lordosis curves were related positively to training time [81]. Moreover, gymnasts showed the greatest spinal curvatures in both types of curves across sports. Those who did not participate in sports had the smallest spinal curvatures. Sex and age did not influence the outcomes [81]. An Australian two-dimensional kinematic study of 122 national team female gymnasts performing a back-bend showed that those with low back pain had slightly greater mobility in the lumbar spine, in combination with a less flexible thoracic spine combined with less flexible hips in extension and hyperextension. However, the difference between the symptomatic and asymptomatic groups was not statistically significant [12]. The investigators also concluded that the period for development of low back pain due to aberrations in spinal curves was prior to the age of 14 years [12].

Extreme ROM among rhythmic gymnasts has always been a main criterion in selection tests and talent identification. Rhythmic gymnasts, as a group, often display extreme spinal ROM, which far exceeds even that of female artistic gymnasts. A specific spinal flexibility test, as described by Galeva in Jemni [82], has been used for more than four decades in countries formerly constituting the Eastern Bloc.

The presence of idiopathic scoliosis was surveyed via a questionnaire in 201 gymnasts, with 192 controls. Those with idiopathic scoliosis were more numerous in the gymnastics group than in the control group, and idiopathic scoliosis was not necessarily present prior to the start of gymnastics. The investigators concluded that there are probably intervening variables in idiopathic scoliosis among gymnasts, such as joint laxity [83]. In a study of idiopathic scoliosis among rhythmic gymnasts, the investigators concluded that there was a dangerous triad of joint laxity, delayed maturity and asymmetric spinal loading, which contributed to the prevalence of this disorder. These rhythmic gymnasts were prepubertal. The authors also postulated that delayed maturation was an additional confounding variable [84]. This health issue, combined with deregulation of the endocrine and reproductive systems, were shown in gymnasts subjected to high training loads [82]. Inheritance may also be a confounding and contributing variable to the incidence of idiopathic scoliosis [85, 86]. Genetic factors may also play a role in talent identification and selection of gymnasts with increased spinal flexibility. Many talent identification tests for gymnasts include assessment of spinal flexibility. Talent identification test items may vary from test to test, but all spinal flexibility items encourage maximum ROM [15, 87–89].

## Joint Hypermobility Syndrome

Joint hypermobility syndrome (JHS) is a medical condition resulting from a genetic anomaly that alters the structure and elasticity of connective tissues. Ehlers–Danlos syndrome and Marfan syndrome are named disorders that are among the family of problems that arise from JHS [7, 26, 27, 90]. As a medical issue, these diseases are syndromes beyond the scope of this review, with the caveat that connective tissue syndromes that permit increased ROM and elasticity may be beneficial to gymnasts and others, but they may require careful management and therapy [28, 91]. Dancers, gymnasts and others requiring large ROMs in their art or sport could be at a clear advantage if they were genetically predisposed to easily acquired flexibility.

Other activities and sports may inform our understanding of hypermobility in gymnastics. Gymnasts perform dance, and many take extensive training in ballet. In a study of the Royal Ballet School in London and 53 student nurse controls, the results indicated that ballet students showed a higher incidence of hypermobility of joints—including the spine, hips and ankles—which would clearly be desirable and influenced by training. However, the dancers also showed greater joint hypermobility in the knees, elbows and wrists, which is not desirable and should not be enhanced by training. Additionally, dancers in the 11- to 15-year age group showed continued retention of joint laxity, while non-dancer controls showed a significant reduction in joint hypermobility scores [26]. Joint hypermobility might appear to be a significant benefit to dancers, but joint laxity in hypermobile dancers was often so great that the dancer could place her body and limbs in unaesthetic positions. Dancers had to learn to consciously limit their excessive ROMs by voluntary muscle control [26]. Dancers also appeared to suffer more ligamentous injury and stress fractures, thus showing that JHS may be a mixed blessing.

JHS would be of benefit to contortionists and some sports [7, 26, 92–94]. However, this syndrome also tends to result in an increased prevalence of osteoarthritis [26], and hypermobility may have a deleterious effect on technique [26]. For example, hypermobile—and thus unstable—elbows would be a significant problem for gymnasts. Gymnastics coaches often comment that they do not desire an athlete who is ‘too flexible’ to the point of near ‘floppiness’, as demonstrated by the athlete’s inability to control his or her limbs in extreme ROM positions [26]. Experience has shown that excessive flexibility of the spine does not result in the same concern about joint stability as knees and shoulders among gymnastics coaches. Moreover, JHS tends to occur in degrees or lie on a continuum from extreme to slight hypermobility. Coaches, parents, physicians and others should proceed carefully with the hypermobile athlete. Hypermobility may need special therapies to assist the athlete in controlling his or her unusual flexibility [28, 91].

Young gymnasts may benefit from initial screening for hypermobility syndrome prior to participation, by use of the Beighton score method and related diagnostic techniques from rheumatology [26, 95, 96]. The Beighton score is derived from observation of:

1. Bilateral knee hyperextension beyond 10° (Fig. 7).

**Fig. 7:**
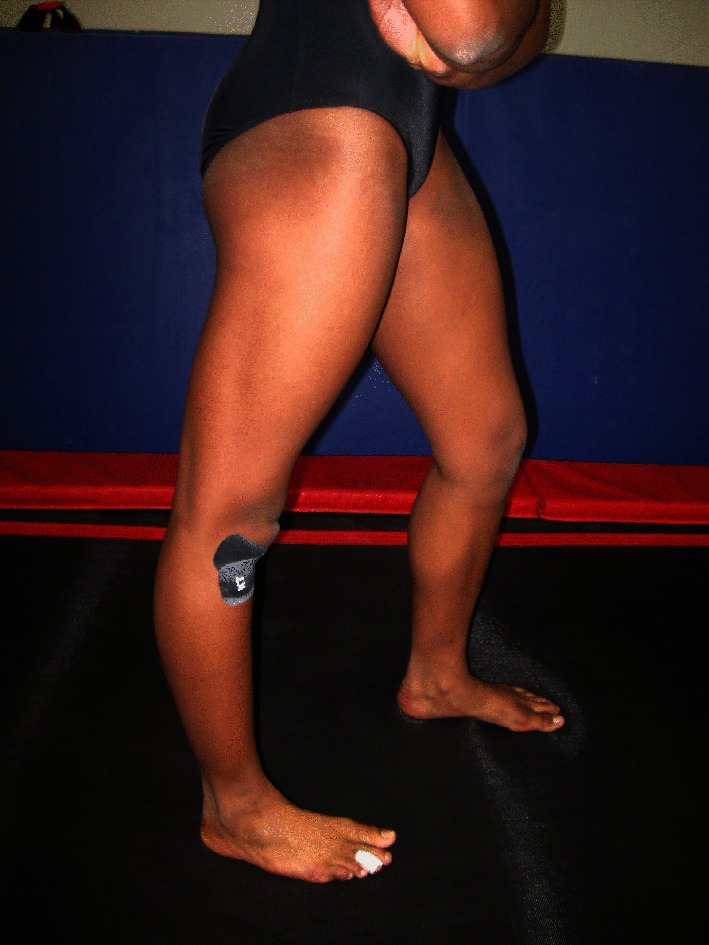
Hyperextended knee
2. Bilateral elbow hyperextension beyond 10° (Fig. 8).

**Fig. 8:**
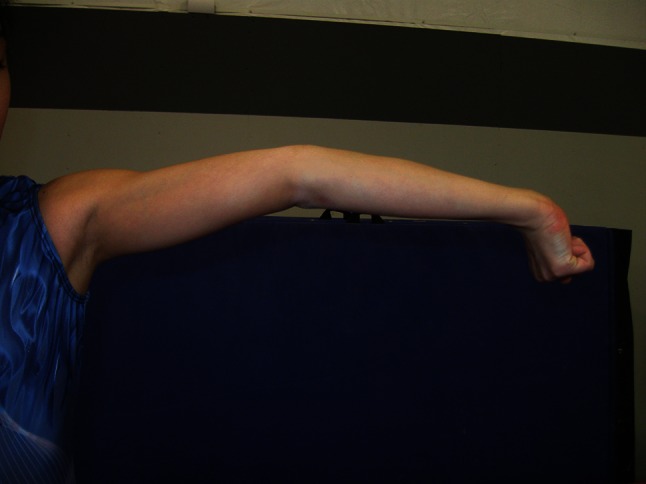
Hyperextended elbow
3. Bilateral flexion of the wrist to touch the thumb to the inside of the forearm (Fig. 9).

**Fig. 9:**
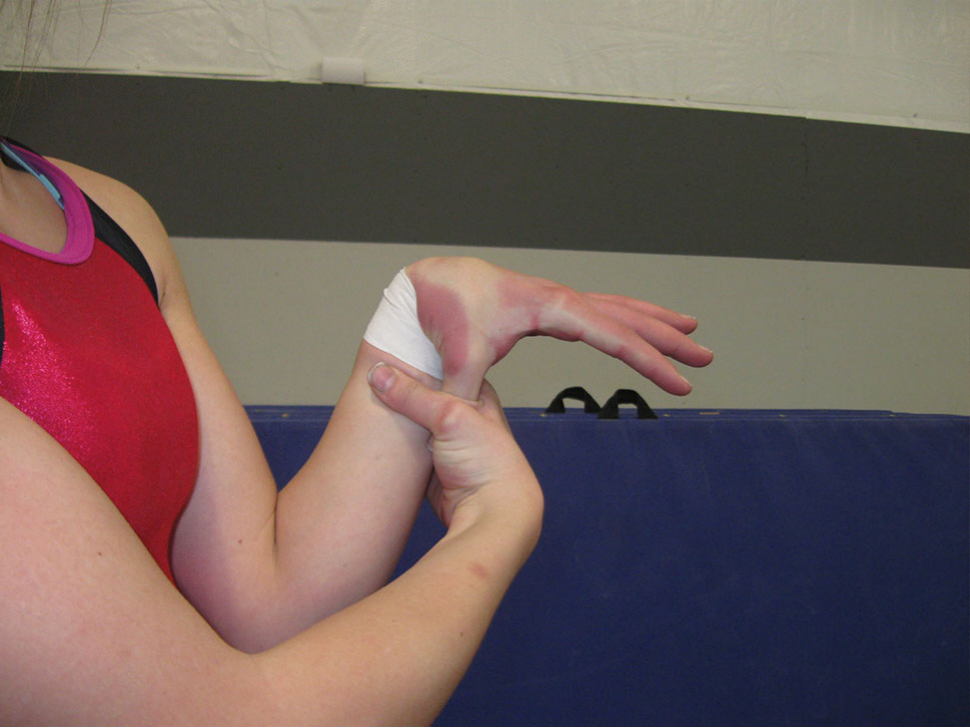
Touching the thumb to the forearm
4. Bilateral ability to place the palm flat on a table and lift the middle or index finger to a vertical position (Fig. 10).

**Fig. 10:**
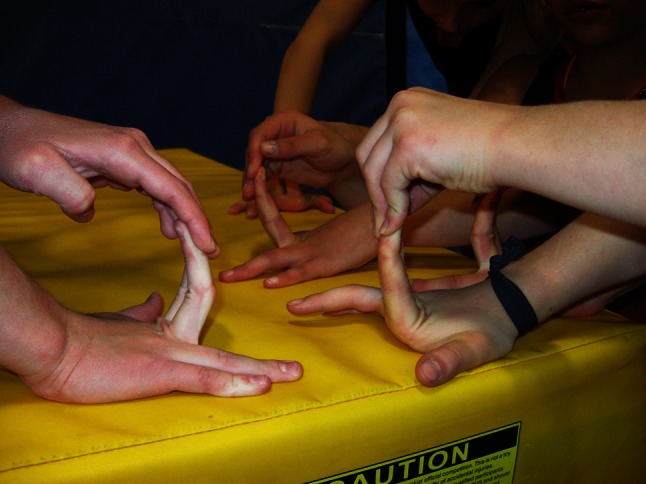
Fingers hyperextended perpendicularly to the hands
5. Ability to palm the floor from a standing ‘toe-touch’ position (Fig. 11).

**Fig. 11:**
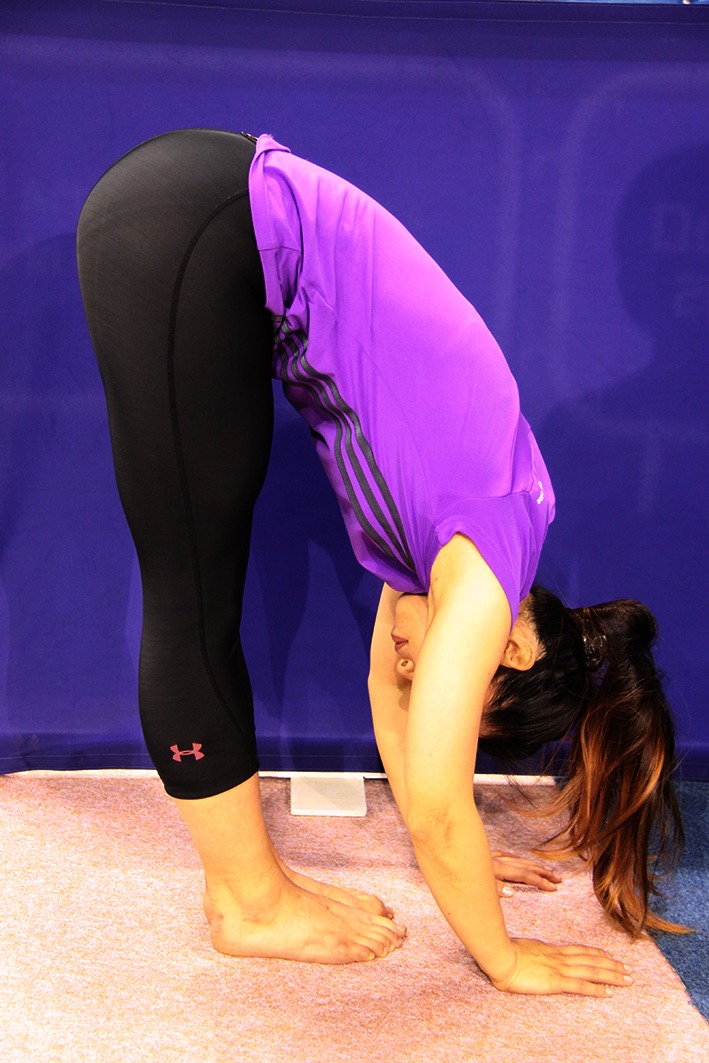
Palms-to-the-floor position

Each position is rated at 1 point if the athlete can achieve the position, and at 0 points if he or she cannot. Thus, a total score of 9 points is possible. If the athlete scores 9 points, then one is wise to suspect JHS, and the athlete should be referred to a physician for further investigation. Although Beighton scoring has been controversial, recent work has shown that a score of 7 points or more identifies approximately 9 % of children as needing additional attention and should be used as the ‘cut-off’ score for determining hypermobility [95]. Unfortunately—in spite of repeated referrals and examples in the JHS literature—to our knowledge, Beighton scoring has not been applied to young gymnasts.

## Contortionism

Contortionists actively practise—and sometimes make their livelihood from—extreme ROMs, particularly of the spine. Contortionists may be an ideal study group for characterizing spinal flexibility in non-gymnasts. Early training and talent identification are common in this group, and the participants are not usually involved in competition [26, 92–94]. Many contortionists appear to have JHS [7]. While these athletes are not using their hypermobility for acrobatics, they achieve many positions that are developed and performed in acrobatics. In 1882, a physician named Owen examined a 34-year-old male contortionist, who could dislocate his hips and shoulders at will and then voluntarily reduce the dislocations. This contortionist had been able to perform feats of extreme flexibility and dislocations since childhood. He was capable of many unusual positions and, according to the accounts of Owen, appeared to be in perfect health [94]. A case study of a 22-year-old Chinese contortionist, involving MRI of the spine, showed a normal spine with mild anterior displacement of L1 relative to L2, mild disc degeneration and anterior spondylosis, mild disc degeneration at T4/5 and no other abnormal findings. The investigator concluded that in spite of extreme ROMs, there were no abnormal subluxations or spinal segmental motions [93]. A case study of a 6-year-old male contortionist showed numerous symptoms indicating JHS, but, in spite of this constellation of symptoms, the youngster was in good health upon examination [97]. Information on contortionists may assist our understanding of gymnasts who perform many of the same movements—albeit usually in less extreme positions and with a different intent—by demonstrating that in spite of flexibility even more extreme than that of gymnastics, contortionists do not appear to suffer from their extreme flexibility.

## Yoga

Yoga is an old art form and exercise system consisting of at least 40 styles [98]. There is a paucity of studies on children and yoga, with studies suffering from low statistical power, varieties of measurements and differing subject characteristics [98]. Studies of yoga and children have been noteworthy because of a lack of reported injurious events [98]. Some styles of yoga include a back-bend position among their postures. The focus of yoga for children is less on postures and more on breathing and breathing relationships to postures [98]. Some forms of yoga require excellent physical condition and may be contraindicated for children lacking the requisite fitness [98]. Young gymnasts, with even a modicum of training, should excel at yoga, given proper instruction. Postural holds are initially of relatively short duration in children aged less than 6 years, usually for a count of ten. Older children may hold postures for 60–90 s. Difficult postures should be followed by a short rest of approximately 15 s. Classes for children’s yoga should be small [98]. In adults, yoga has been shown to reduce some pain and disability. For example, relief of idiopathic and degenerative scoliosis was obtained following 6 months of practising the yoga plank exercise [99]. A review of Iyengar yoga also found evidence of back and neck pain relief [100].

Hold times for yoga postures are similar to those assigned to gymnastics stretching exercise positions, other than back-bends [22, 101]. In gymnastics, back-bends are usually held for only a few seconds before descent to a supine position or movement to new position [54]. The back-bend posture has found its way into both yoga and gymnastics exercises. Interestingly, yoga uses a back-bend for therapeutic purposes and enhancement of health, while gymnastics tends to use the back-bend as a means to learn and perform other skills in competition.

## Spinal Injury

Tissue damage and pain from spine stretching demands analysis against the background of ‘typical’ spine pain and injury acquired without intensive stretching. Previous symptoms of spine pain may be the best predictors of later spine pain [5]. Children usually present with an identifiable structural cause—as opposed to adults, who present with more vague symptoms, which may not be evident on conventional imaging [5]. Thus, provocative testing and spine imaging are particularly important in children [5, 16]. Generally, pain on flexion usually indicates an intervertebral disc source, while pain on extension and hyperextension usually indicates anomalies in posterior structures [5, 16].

A study of highly trained female gymnasts and swimmers showed that gymnasts had a greater incidence of spinal abnormalities that were correlated with training hours [20]. The background incidence of back pain in children is approximately 18 %, with athletic children showing incidences of 46 % and gymnastics literature indicating ranges from 11 to 85 % [5].

Given that there is almost no information on spinal injury and early childhood gymnastics, one is forced to assess somewhat older children and their spine complications. Inference of findings in older gymnasts to their younger counterparts is fraught with a number of threats to the validity of conclusions. Among these problems is an inability to account for long-term changes in gymnasts that are due to diet, home cultures, training loads, changes in gymnastics culture and many others. However, one can likely assume that symptomatic spine problems arising in late childhood, early adolescence and young adulthood had their origins at younger ages. A comparison study of injury symptoms and flexibility was performed, which involved female gymnasts (*n* = 60) and an age-matched control group (*n* = 35) aged 5–17 years [6]. More gymnasts than controls had injury symptoms in the wrist, low back, hip, shin and foot. Gymnasts had an average of 6.17 symptomatic regions, versus 2.25 in controls. Gymnasts had greater shoulder flexion, horizontal abduction, lumbar flexion, hip extension and toe-touching abilities. Controls had better forearm supination. No statistical differences were found between the groups in terms of ROM of the lumbar spine, knee or elbow extension [6]. No statistically significant correlations were observed between different body regions in ROM. Gymnasts with sore backs had greater toe-touching ability; however, no consistent or significant relationships between ROM in a body region and injury were found [6]. A 1991 study comparing gymnasts and swimmers showed that 9 % of pre-elite, 43 % of elite and 63 % of Olympic gymnasts had spinal abnormalities, versus only 15.8 % of swimmers [20]. The spine and other parts of the body also presented interesting paradoxes by showing structural abnormalities without pain symptoms. However, much older male gymnasts (aged 19–29 years) were examined by MRI and showed significant correlations between lumbar spine pain and disc degeneration [102]. Goldstein et al. [20] also suggested that training for more than 15 h per week resulted in increased degenerative changes in the lumbar spine. Injuries to the thoracic spine, although rare, have been observed in gymnasts [36, 103]. An epidemiological, multi-year study of stress fractures found that females had more stress fractures, female gymnasts ranked second behind cross-country runners and spine stress fractures ranked third in prevalence by body region [104]. Gymnastics spinal injuries have been a noted area of concern, with aetiologies involving a number of injury symptoms and mechanisms [105, 106].

Injury patterns in gymnastics do not appear to conclusively indict spinal flexibility alone as a precursor to injury. Gymnastics combines high forces of motion and impact with an unstable landing position or fall [53, 107–111]. Landing energies in gymnastics range from 1500 to 2200 N∙m [112] and from 14 to 18 times body weight [111, 113]. The combination of high forces and high repetitions is a likely mechanism for spinal injuries [114]. An epidemiological injury study showed that 70 % of injuries could be predicted by knowing the subject’s weight, height, mesomorphy, lumbar posture and age [115]. In a 1985 study, Ciullo and Jackson [116] postulated that repetitive hyperextension and microtrauma of the spine were potential causes of spinal injury. Radiological findings in preadolescent and adolescent female gymnasts showed that one third of the gymnasts had spinal abnormalities classified as severe and another third had abnormalities classified as moderate [36]. Among younger (9- to 13-year-old) female gymnasts, the water content and disc height of the intervertebral discs were greater than those in a control group [36]. Another study indicated that genetic predisposition was probably the major determinant of spinal disc degeneration [117]. An MRI study of 35 preadolescent gymnasts showed that only three had observable disc degeneration. The authors concluded that in spite of excessive ROMs and large axial loading of the spine, primary damage to intervertebral discs was uncommon in young gymnasts [80]. Similar findings in Olympic-level gymnasts aged 12–20 years were found by Bennett et al [118].

A study of rhythmic gymnasts aged 13–19 years showed that youth, greater leanness, non-smoking, less anxious or depressive behaviour, and increased muscle strength and flexibility all represented preventive factors for low back pain. This study suggested that rhythmic gymnastics did not increase the risk of low back pain [119]. In contrast to conventional wisdom, retired rhythmic gymnasts did not show an increased incidence of low back pain when compared with age-matched controls [120]. However, those rhythmic gymnasts who had low back pain while competing were more likely to cease participation and had an increased risk of low back pain following retirement [120].

## Risk Management and Countermeasures

Performing a back-bend requires specialized fitness. Teaching a back-bend requires sound coaching judgment and serious attention to detail. Only those athletes who are ready for this skill should attempt it. Great care should be exercised to ensure that coaches monitor technique and fatigue in all skills. Coach education should be a high priority for all those engaged in teaching the back-bend. On the basis of a lack of a clear consensus on the danger of the back-bend, the lack of clear epidemiological trends and relationships, and decades of experience with the skill, it is probably a safe skill for most youngsters. According to Beighton et al. [26], “Individuals must be considered on their own merits according to their sport, and different joints within the same person are likely to respond to different training programmes”. Considerations that are likely to be important in sound coaching judgment can be found in the following extract from Armiger and Martyn [121]: “Our flexibility potential is affected by genetics, gender, age, lifestyle, medical history, occupation, and of course, type and level of physical activity. It is therefore unwise to assume that all stretches are beneficial and safe for everyone”.

As a guideline for dealing with an athlete who struggles with spinal flexibility, consider that young gymnasts complaining of back pain should never be ignored or assumed to be suffering from a simple back strain, muscle spasms or ‘growing pains’ [122]. Persistent back pain that lasts longer than 2 weeks should result in referral of the gymnast for a complete evaluation, a careful medical history, and a four-view radiographic assessment, bone scans or other advanced medical techniques, as dictated by a licensed sports medicine physician [123]. Finally, coaches should carefully observe where the athlete actually bends. The ‘hinge’ point may be inadvertently placed in the lumbar spine because of a lack of flexibility of the thoracic spine and hip flexors. Unfortunately, stiffness in these areas often goes unnoticed until pain symptoms appear. Inclusion of specialized stretching to enhance thoracic and hip extension and hyperextension ROM may make specialized stretching a saviour of the gymnast’s lower back rather than a contributor to low back pain.

## Conclusion

Although the research literature does not provide a clear consensus on the safety—or lack thereof—of the back-bend in young children, the literature also does not condemn the skill as too dangerous. Clearly, more research needs to be conducted. However, the back-bend does not appear to provide a threat to the health of youngsters, provided that they are well supervised, are carefully instructed through lead-up skills, possess the strength to support themselves in the position and understand that if they feel pain, they must contact their coach immediately so that the pain can be assessed. While all sport skills present some risks, the back-bend position appears to present minimal risk. One should be able to accommodate the needs of the young gymnast through simple sound and vigilant coaching judgment.
